# MRI in T staging of rectal cancer: How effective is it?

**DOI:** 10.4103/0971-3026.63055

**Published:** 2010-05

**Authors:** MG Mulla, R Deb, R Singh

**Affiliations:** Royal Derby Hospital, Derby, UK

**Keywords:** Rectal cancer, MRI, staging

## Abstract

**Background:**

Rectal cancer constitutes about one-third of all gastrointestinal (GI) tract tumors. Because of the high recurrence rates (30%) in rectal cancer, it is vitally important to accurately stage these tumours preoperatively so that appropriate surgical resection can be undertaken. MRI is the ideal technique for the preoperative staging of these tumours.

**Aim:**

To determine the accuracy of local T staging of rectal cancer with MRI, using histopathological staging as the gold.

**Materials and Methods:**

Forty consecutive patients admitted with rectal cancer over a period of 18 months were included in this retrospective study. MRI scans were performed prior to surgery in all patients, on 1.5T scanners. Two radiologists, with a special interest in gastrointestinal imaging reported all images. Two dedicated histopathologists reported the histology slides. The accuracy of preoperative local MRI T staging was assessed by comparison with postoperative histopathological staging.

**Results:**

There was agreement between MRI and histopathology (TNM) staging in 12 patients (30%). The sensitivity and specificity of MRI for T staging was 89% and 67% respectively. The circumferential resection margin (CRM) status was accurately staged in 94.1% of the patients.

**Conclusions:**

Preoperative staging with MRI is sensitive in identifying CRM involvement, which is the main factor affecting the outcome of surgery.

## Introduction

Rectal cancer is a common tumor in the Western world and is one of the most common malignant tumours of the gastrointestinal (GI) tract.[1] More than 14,000 new cases are diagnosed every year in the UK. The higher prevalence in the West as compared to the developing world has been attributed to differences in diet.[2]

**Figure 1:**
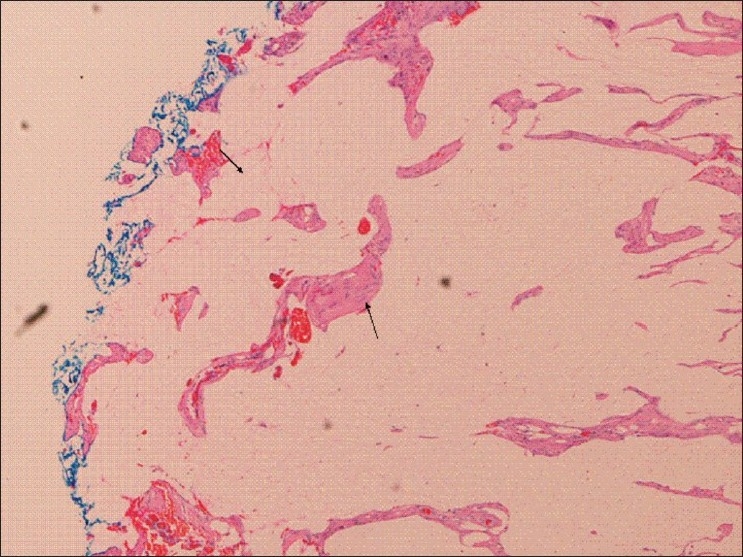
Histology (H &E) showing CRM (left arrow) and its involvement by tumour (right arrow)

The disease is more common after the age of 50 and shows a slight male predilection. Over the last decade, many improvements have been made in the management of rectal cancer. With better radiological staging, curative surgical resection is becoming more popular. The recurrence rates after surgery vary from 3 to 32%.[3–5] Local tumour spread, involvement of lymph nodes, and distant metastases all influence the prognosis of rectal cancer.[6,7] There is an increasing need for accurate preoperative staging because aggressive multimodality treatment approaches are being employed these days based on individual risk factors.[8,9] Histopathologic tumour involvement of the circumferential resection margin (CRM), which is the peritoneal reflection of the mesorectal fascia [Figure 1] has been shown to be an independent predictor of local recurrence and, hence, influences overall survival after primary resection.[10,11] MRI is a promising tool for staging rectal cancer preoperatively and can also provide measurements of the distance to the mesorectal fascia, which forms the potential resection margin in total mesorectal excision.[12]

## Materials and Methods

Forty consecutive patients with a histologically confirmed diagnosis of rectal cancer, admitted to our hospital over a period of 18 months, were included in this retrospective study. Patients who had received chemotherapy and/or radiotherapy were excluded from the study to avoid errors in staging and analysis of results.

All patients were staged with MRI preoperatively. All images were reported by two radiologists with a special interest in colorectal imaging. Histology slides from the resected specimens were reported by two dedicated histopathologists with special interest in colorectal cancer staging. TNM staging was done as shown in Table 1.

**Table 1:** TNM staging of rectal cancer

Stage	Level of involvement
Tumor	
T1	Limited to mucosa and submucosa
T2	Extension into but not through muscularis propria
T3	Invasion of perirectal fat
T4	Invasion of adjacent structures
Nodes	
N0	No involved lymph nodes
N1	Fewer than four regional nodes involved
N2	More than four regional nodes involved
N3	Distant nodes involved
Metastasis	
M0	No metastasis
M1	Distant metastasis

### MRI technique

All the scans were performed on one of two 1.5T scanners (Signa Excite, GE Medical Systems, Milwaukee, USA or Philips Intera, Philips Medical Systems, Eindhoven, the Netherlands) with an 8-channel cardiac coil (GE scanner) or a synergy body coil (Philips scanner). The scans were supervised by the reporting radiologists who planned the angled axials (perpendicular to the tumor bulk) and coronal (parallel to the anal canal) images, where needed. The sequences used were T2W sagittal (3 mm), T2W axial (angled, 3 mm), and T2W coronal (for low rectal cancers, 3 mm).

An additional axial T2W sequence through the pelvis, with a larger field of view (slice thickness: 6 mm), was performed up to the iliac crest for identifying nodal disease. All T2W sequences were non-fat-suppressed. The typical TR and TE values were as shown below [Table 2]:

**Table 2:** MRI technique -TR and TE values

	Philips Intera^®^ 1.5 Tesla	GE Signa Excite^®^ 1.5 Tesla
TR	3500	3620
TE	90	106.44

## Results

Our study sample included 25 males and 15 females with a median age of 70.5 years. We assessed agreement between MRI and histopathology for T staging by calculating the kappa coefficient (k value) which was 0.458, which demonstrates moderate agreement. The sensitivity and specificity of MRI for T staging were 89% and 67% respectively. The positive predictive value (PPV) and negative predictive value (NPV) of MRI for T staging were 85% and 15% respectively.

**Figure 2:**
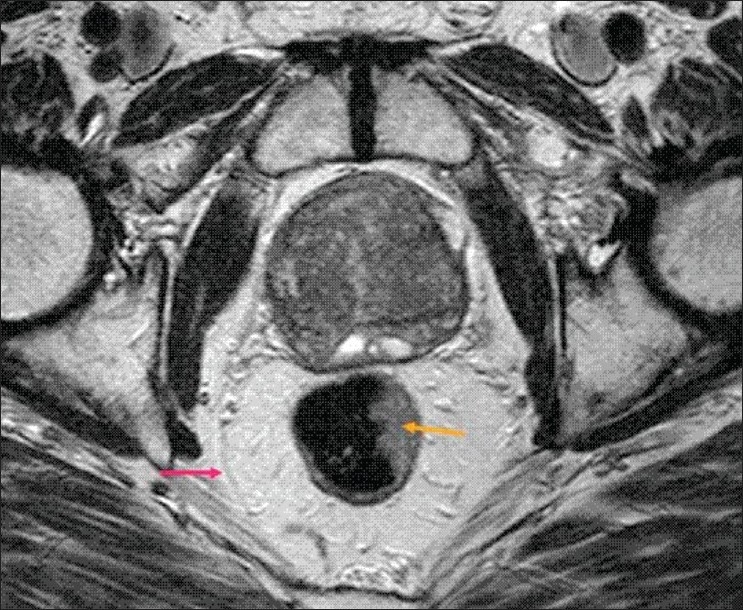
MRI echo T2 w spin echo image shows CRM - peritoneal reflection (arrow pink). Note the eccentric left lateral wall tumour (yellow arrow). Note the CRM is not involved in this case

**Figure 3:**
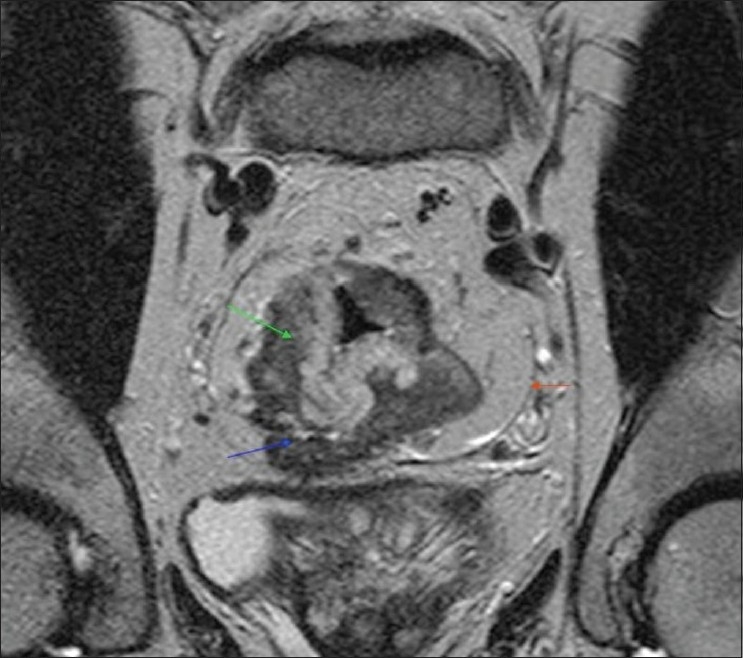
Coronal T2 spin echo MRI showing bulky rectal tumor (green arrow), CRM (red arrow) and its involvement by the tumor (blue arrow)

In 21 patients, T staging with MRI did not match with histological staging. Of these, 17 mismatches were between T2 and T3 stages. The CRM status was assessed further in these 17 patients as this is clinically very important for further decision making. The CRM was found to be accurately staged [Figures 2 and 3] in all except one patient out of 17 (94.1%). The one false-negative case was due to the difficulty in identifying the CRM. The remaining four mismatches were between T3 and T4. All these four cases had perforated tumours on histology, which explains their being under-staged on MRI.

## Discussion

MRI scan is one of the tools used in the evaluation of rectal pathologies. Its anatomic location, its fixation in the pelvic fat, and the lack of peristalsis make the rectum an ideal organ for imaging with MRI.[13] This modality has been invaluable in the diagnosis and staging of rectal cancers. Although rectal tumours can be diagnosed with digital examination, barium enema, and colonoscopy or sigmoidoscopy, these endoluminal techniques do not provide sufficient information about the extraluminal spread of the tumour, which is necessary for preoperative planning.[14] With the better soft tissue contrast provided by MRI as compared to CT, it is the recommended modality of investigation, especially for low-lying rectal tumours.[15] The mesorectal fascia, which is the border for total mesorectal excision (TME), is clearly seen on MRI.[16] MRI is also helpful for differentiating early recurrence from postoperative changes and for the evaluation of perianal fistulas and sinus tracts.[17,18]

In other similar series the overall weighted agreement between MRI and histology for T staging has ranged from 66–94%.[19,20] The main difficulty with MRI has been in the differentiation between T2 and T3 tumours. However, with the definitive treatment being the same for both these stages, this differentiation does not impact hugely the patient outcome.

The accuracy of MRI for detection of CRM involvement was 94% in our study, with only one patient being wrongly staged. The reason for this could be the difficulty in interpretation of the CRM on MRI which is a well known problem. CRM involvement still remains the single most important factor in predicting the prognosis of rectal cancer[19]

The percentage of CRM-positive patients has varied in different series. It is dependent on patient selection, performance of preoperative imaging, preoperative therapy, surgical technique, and the skill of the pathologist.[21] However, results from nine studies, involving a total of 529 patients, have shown that MRI has an overall sensitivity and specificity of 94% and 85%, respectively, for detecting CRM involvement preoperatively.[22] These percentages indicate that MRI is very sensitive in predicting CRM involvement, which information is essential when formulating the treatment in individual cases.

Endorectal USG is a modality that is becoming increasing popular and is considered an equally suitable imaging technique for the initial staging of rectal cancer.[23] It has the ability to demonstrate the different layers of the rectal wall (mucosa-muscularis mucosae, submucosa, and muscularis propria) and is, therefore, generally quite accurate, both in evaluating the early stages (T1 and T2) and in demonstrating the perirectal spread of cancer (T3). However, endorectal USG has limitations in the evaluation of the mesorectum and its fascia; also, it cannot be used in highly stenosing tumors due to difficult access.[24,25]

Compared to endorectal USG, MRI is more accurate for the evaluation of the mesorectum and the mesorectal fascia, involvement of which are considered the most relevant prognostic factors for local recurrence after the introduction of standard total mesorectal excision.[26] MRI is also considered the most reliable tool for demonstrating tumor invasion of surrounding viscera (T4) because it provides a large field of view, multiplanar vision, and superb contrast resolution.[27] Combined endorectal and pelvic phased-array coil MRI can be used reliably and are highly predictive in T3 tumors but have limitations in assessing lymph nodes.[28]

## Conclusion

Preoperative staging with MRI is very sensitive in identifying CRM involvement, which is the main factor affecting the outcome of surgery. There are still problems in differentiating T1 from T2 as the lesions at these stages are limited to the mucosa and muscularis propia and hence are difficult to delineate.
